# The Impact of 3D Printing Parameters on the Post-Buckling Behavior of Thin-Walled Structures

**DOI:** 10.3390/ma13214742

**Published:** 2020-10-23

**Authors:** Tomasz Kopecki, Przemysław Mazurek, Łukasz Święch

**Affiliations:** Faculty of Mechanical Engineering and Aeronautics, Rzeszów University of Technology, al. Powst. Warszawy 8, 35-959 Rzeszow, Poland; tkopecki@prz.edu.pl (T.K.); pmazurek@prz.edu.pl (P.M.)

**Keywords:** aircraft load-bearing structures, finite element method, nonlinear numerical analyses, stability, equilibrium path, 3D printing, digital image correlation

## Abstract

This study presents the results of experimental research and numerical calculations regarding models of a typical torsion box fragment, which is a common thin-walled load-bearing structure used in aviation technology. A fragment of this structure corresponding to the spar wall was made using 3D printing. The examined system was subjected to twisting and underwent post-critical deformation. The research was aimed at determining the influence of the printing direction of the structure’s individual layers on the system stiffness. The experimental phase was supplemented by nonlinear numerical analyses of the models of the studied systems, taking into account the details of the structure mapping using the laminate concept. The purpose of the calculations was to determine the usefulness of the adopted method for modeling the examined structures by assessing the compliance of numerical solutions with the results of the experiment.

## 1. Introduction

In aviation technology, due to the very specific nature of the considered objects, certain standards that affect the design processes and operational assumptions have been formed. One of the principles relating to the most commonly used metal structures in aviation allows for the admission of the post-critical deformation of selected types of structures in the operational load ranges [1,2].

In general, due to the necessity of minimizing the mass of an object, the loss of stability of the skin under operating conditions is permissible, as long as this phenomenon has an elastic character and occurs locally, i.e., within the segment of the skin and limited by skeletal elements [3,4]. In the case of metal structures, such a state is achieved through the use of thin-walled skins, which are often reinforced with a number of stiffening elements [5,6,7,8].

The problem of the loss of stability of thin-walled load-bearing components has been widely examined for structures based on isotropic materials, such as metals. However, for different material solutions, such as composites or printed structures, the mentioned phenomenon strongly depends on many additional factors and constitutes a new problem, which must be precisely described with the results of suitable research, in order to fulfill all demands and regulations related to the creation of aircraft structures.

At the present time, there is also a tendency to replace traditional metal structures with construction elements made of materials that enable them to be shaped more freely [9,10]. In addition to layered composites based on epoxy resins and glass, carbon, or aramid fabrics, the planning phase of design processes is increasingly taking into account solutions using 3D printing [11,12]. This avoids troublesome and costly technological processes, such as plastic working, machining of metal structures, or complex heat treatments for composites [13,14].

Although the use of printed components in aircraft structures is still very limited, it should be expected that this situation will soon change. This is due to both the minimization of production costs and the enormous progress made in improving the properties of construction materials used for printing, as well as of the printing devices themselves. Currently, there are many applications of this technology for the production of small unmanned aerial vehicles.

In this situation, it seems advisable to conduct research on the properties of various construction solutions for thin-walled load-bearing structures used in aviation technology that are produced with 3D printing.

In the case of full-size aircrafts used for the transport of passengers or goods, printing technologies using light metals or plastics with special mechanical properties are a natural choice [15]. However, it should be emphasized that this area of modern aviation technology also includes miniature aircrafts that perform a whole spectrum of functions, ranging from civilian to military purposes, e.g., inspection or reconnaissance activities [16,17,18,19,20,21]. Devices of this type, which have relatively small dimensions, are generally of relatively low weight; therefore, their structures can be based on materials of much lower strength. One such material that can be used in the construction of these aircrafts is polylactic acid (PLA). This material, which is fully biodegradable and obtained from renewable raw materials such as cornmeal, has found applications in various fields of technology, particularly in biomedical technology. Currently, it is one of the cheapest and most popular materials used for forming objects through 3D printing. The possibility of its use in aviation technology is due to its low mass (density = 1.21–1.43 g cm^−3^) and relatively favorable strength properties.

The use of PLA as a construction material for autonomous or remotely controlled aircrafts moving within the spaces supervised by flight control authorities requires the development of calculation methods that allow one to obtain and document the strength properties required by the regulations, while at the same time obtaining the desired object mass. On the other hand, construction calculations conducted with the use of modern engineering tools, such as software based on the finite element method, require precise modeling of the mechanical properties of the material, not only by using appropriate physical constants, but also by correctly mapping the structures of load-bearing objects.

**Figure 1:**
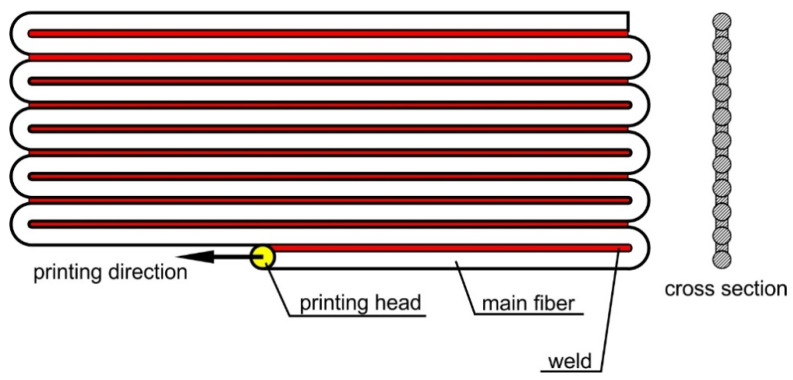
Diagram of the structure of a single layer made using the 3D printing technique.

In the case of 3D printing, as its involves the application of successive layers of polymer, the structure of the obtained system is not consistent and such materials cannot be treated as isotropic. The method of creating a single layer during printing is reduced to the formation of interconnected rows of material fibers (Figure 1). The layer, therefore, has orthotropic properties. In the case of solid objects consisting of a large number of layers arranged in different directions, it can be concluded that the resulting structure has mechanical properties similar to isotropic properties. The situation is different in the case of thin-walled structures composed of just a few layers. In this case, the orientations of the fibers in individual layers have a significant impact on the nature and size of the system’s deformation. In this case, the numerical representation of the structure requires it to be treated as a type of laminate.

Another problem is the correct determination of the mechanical properties of the material; although the available publications contain information on the physical constants required for numerical modeling [22,23], it should be noted that the properties of a single layer of a printed structure depend not only on the printing direction, but also on the individual features of the printing device, the parameters controlling the printing process, and even the individual properties of a given batch of raw materials [24]. Therefore, it seems justified to determine the mechanical properties of the modeled object’s material through appropriate experiments.

This paper presents the results of comparative experimental studies of a typical fragment of an aircraft load-bearing structure, operating in the range of post-critical loads and undergoing large deformations. The main aim of the experiment was to determine the influence of the printing direction of individual layers of the structure on the size of the structure deformation.

It should be underscored that there are numerous factors that can determine the properties of the printed structures. First of all, some technological parameters related to the quality of the object should be mentioned, such as temperature, printing speed, filling strategy, or the height and width of individual layers. However, if all of these parameters are the same for all of the examined structures, the printing direction becomes the parameter that has the most significant influence on the post-critical deformation forms.

Numerical calculations of a nonlinear nature were also performed using commercial software based on the finite element method, with the aim of defining an effective modeling technique for the considered objects. The mechanical properties of the material were determined experimentally using a universal testing machine and a dedicated test stand.

## 2. Materials and Methods

**Figure 2:**
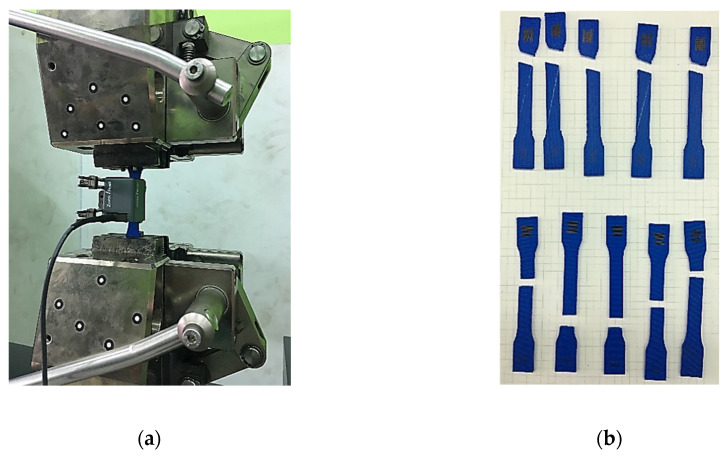
(**a**) View of the sample with the extensometer on the test stand. (**b**) A set of samples with longitudinal and transversal fiber orientations after completion of the test.

The main factor that enables reliable results to be obtained for the numerical calculations is the correct representation of the mechanical properties of the material. The basic step in the presented research cycle was to determine the values of the appropriate physical constants for the printed material. First of all, the values of Young’s modulus and the tensile strength were determined for samples with longitudinal and transversal fiber orientations. The experiment was conducted using the Zwick-Roell Z050 universal testing machine (Zwick-Roell GmbH and Co. KG, Ulm, Germany) and an incremental clip-on extensometer (Figure 2).

**Figure 3:**
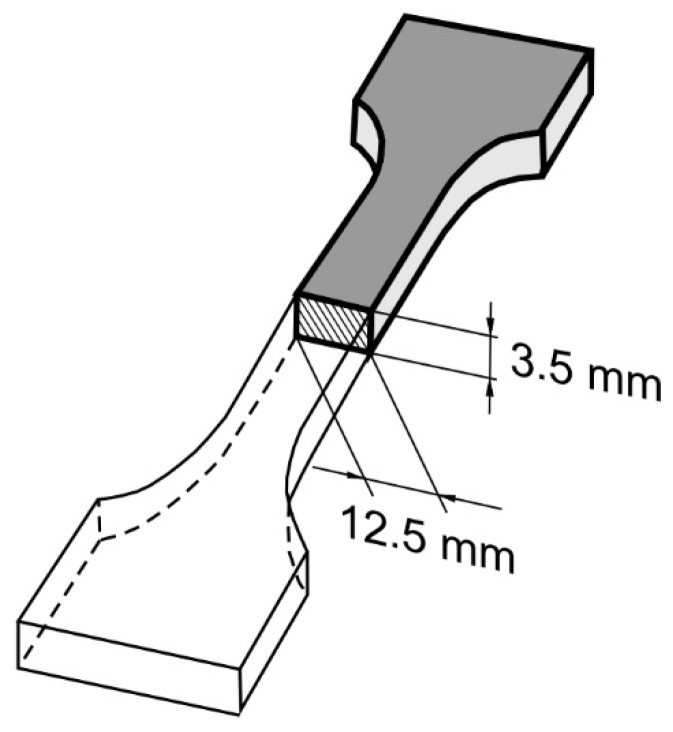
Scheme of the sample with the cross-section dimensions.

Printed samples with a typical geometry based on ASTM D638-14 [25] type 3 specimens, with cross-section dimensions resulting from the selection of print parameters, were used for the experiment (Figure 3). The specimens were prepared with the use of Simlify3D commercial slicer software (Simlify3D, Cincinnati, OH, USA) and manufactured with the use of a Leapfrog Creatr Dual 3D printer (Leapfrog 3D Printers, Alphen aan den Rijn, The Netherlands).

In the next step, the Poisson number was measured. For this, a sample with a longitudinal fiber orientation was elongated. Measurements were made using the Aramis optical scanner (GOM GmbH, Braunschweig, Germany) based on the Digital Image Correlation method (DIC) [26]. This method enables noncontact measurements of the displacement field through analysis of the changes in the distribution of stochastically distributed spots in the photos of the sample in the subsequent loading phases [27,28,29].

**Figure 4:**
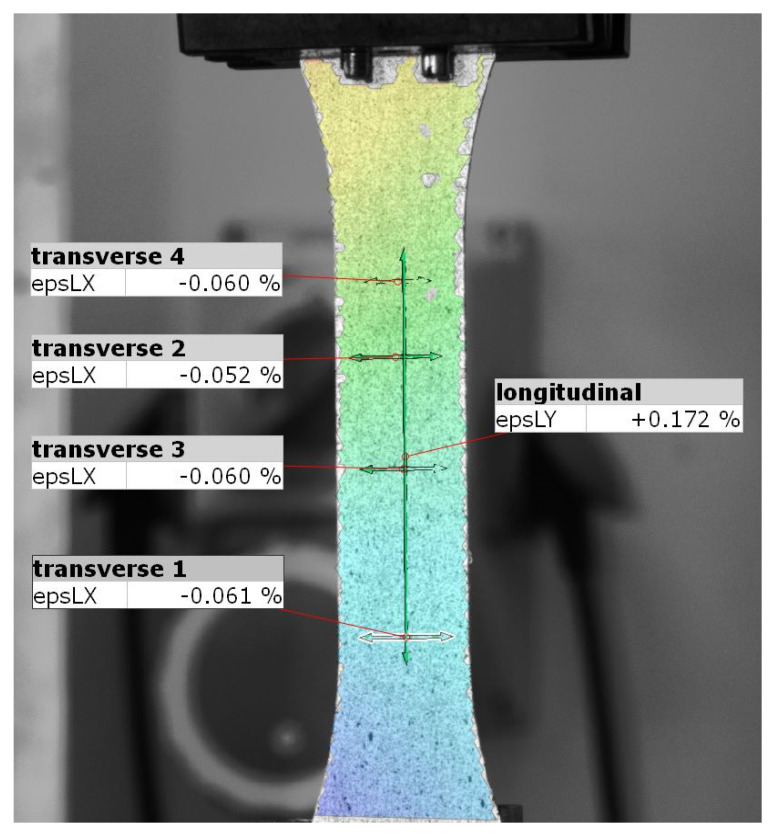
Example of “virtual extensometers” generated with an optical scanner.

The device software allowed for the creation of five “virtual extensometers” (Figure 4), the indications of which corresponded to the precise measurement of the displacement of the limit points [30]. Thanks to these, it was possible to determine the relationship between the elongation of the selected vertical section and the shortening of the horizontal sections. For each of the indications, the equation of a straight line was determined with the least squares method. Application of the DIC method for the tensile test was successfully used in previous research studies related to 3D printed elements [31,32,33].

The last step of the preliminary research phase was to determine the shear modulus for a printed material with a unidirectional fiber orientation. It was considered that due to the orthotropic nature of the system, this constant cannot be determined in accordance with the formula corresponding to isotropic materials.

**Figure 5:**
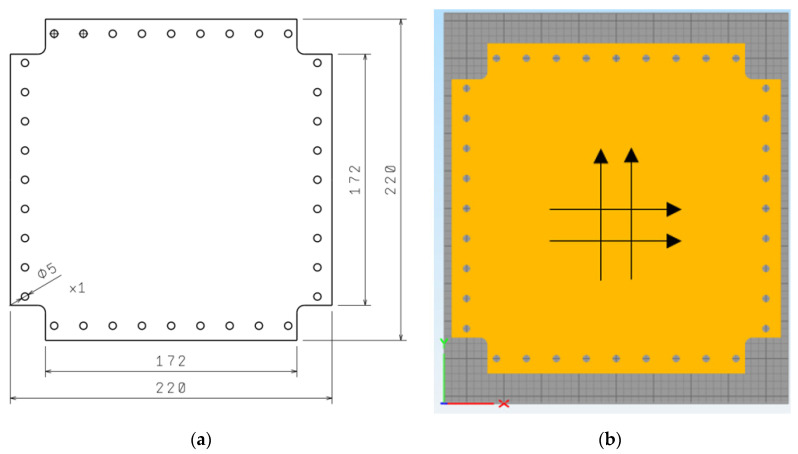
Dimensions (**a**) and printing direction diagram (**b**) for the shear modulus sample.

Due to the predicted even number of print layers in the target spar wall model with alternate vertical and horizontal fiber orientations, a 1 mm thick printed square plate composed of 10 layers was used to determine the shear modulus (Figure 5). The print width, resulting from the diameter of the nozzle applying the material, was 0.4 mm.

**Figure 6:**
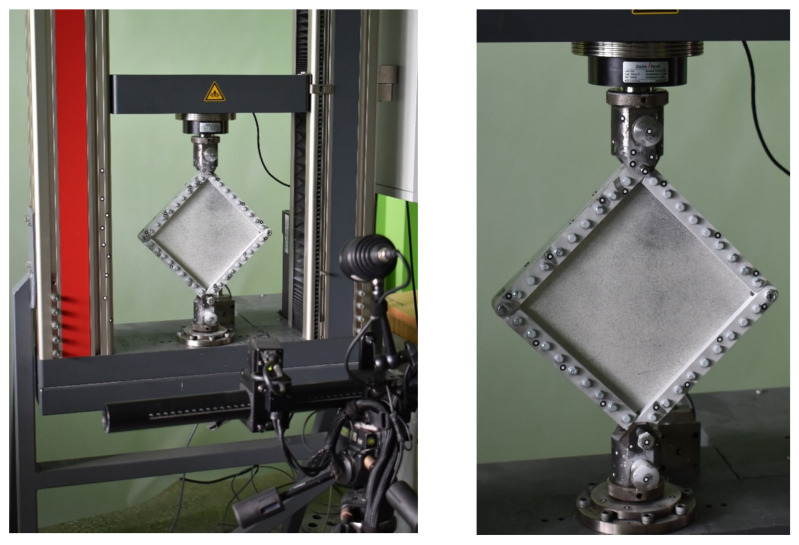
Shear modulus measurement stand.

The experiment was conducted using a special device consisting of four double steel beams, which were articulated at the corners (Figure 6). The edges of the plate were fixed between the beam stiffeners with bolted connections. Two opposite corners of the device were connected to knots fixed in the jaws of the testing machine.

The plate, fixed in the presented way, was subjected to a pure shear. The value of the shear modulus was determined based on Equation (1):

(1) $$\gamma =\frac{\tau }{G}\Rightarrow \;G=\frac{\tau }{\gamma },$$

**Figure 7:**
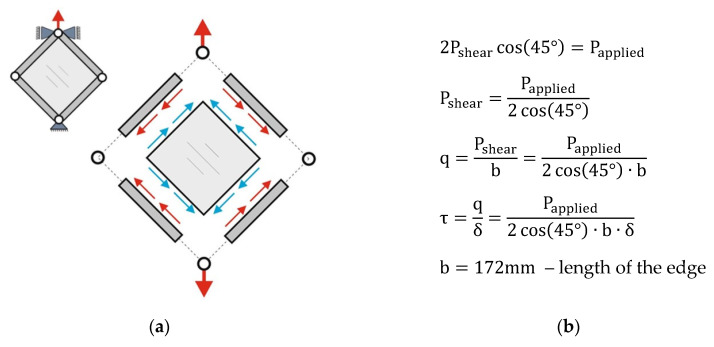
Scheme of the plate load (**a**) and the procedure for determining the tangential stress value (**b**).

It was, therefore, necessary to determine the tangential stress and the shear strain angle. The value of the tangential stress was determined based on the measured value of the tensile force (Figure 7).

**Figure 8:**
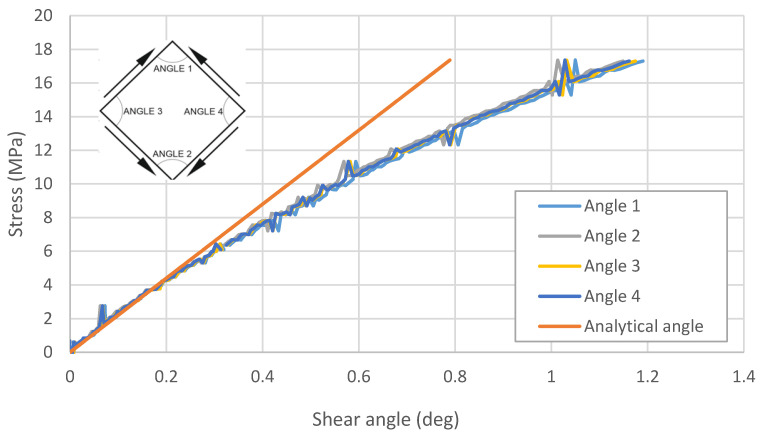
Relationship between the measured values of the shear strain angles and the value of the stress in the plate. The analytical angle was designated based on the value presented in Figure 9.

On the other hand, the shear strain angle was determined based on the measurements with the Aramis optical scanner. During the experiment, the relationship between the values of the angles between the plate’s edges and the value of the stress was determined (Figure 8).

**Figure 9:**
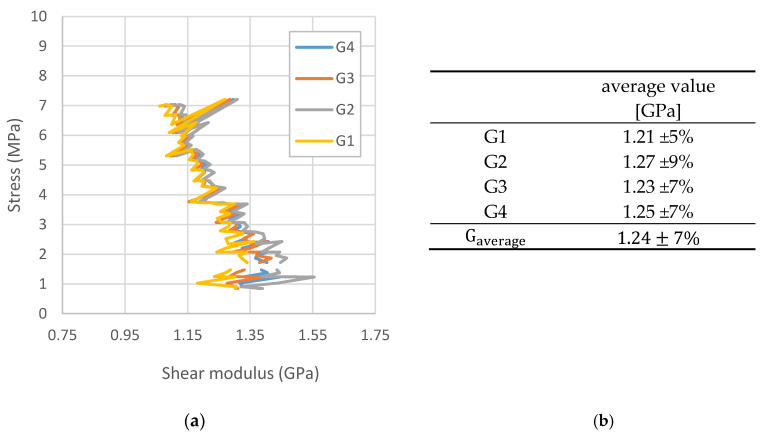
Determination of the average value of the shear modulus.

On the other hand, for each of the shear strain angles, based on Equation (1), the average values of the shear modulus were marked and its final average value was determined (Figure 9).

**Figure 10:**
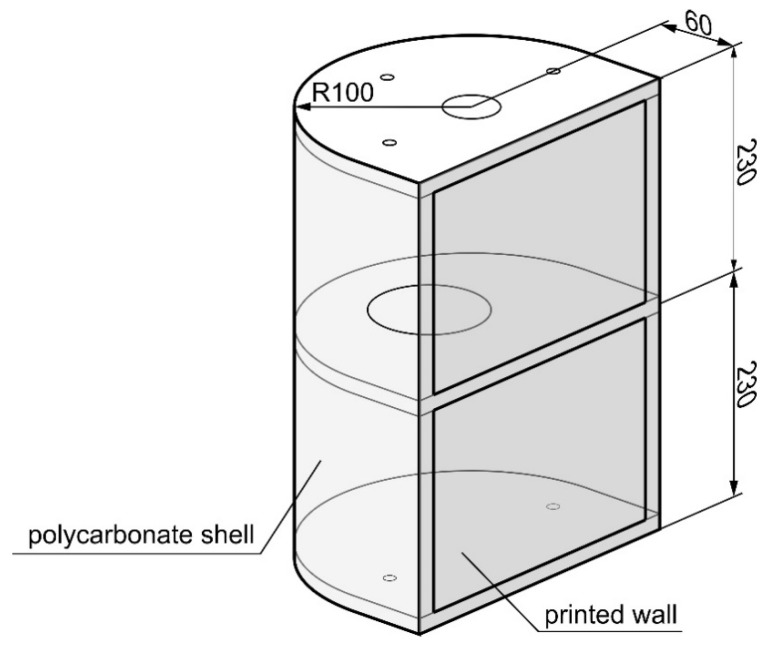
Schematic diagram of the torsion box model.

The subject of the next fundamental phase of the experimental research was the thin-walled air structure model (Figure 10).

Both ribs and the skin of the torsion box were made of polycarbonate under the trade name Makrolon (Covestro AG, Leverkusen, Germany), which is a material that can be considered isotropic due to the random arrangement of its polymer chains. This was confirmed by tests on material samples, which also proved that polycarbonate keeps its elastic characteristics across a sufficiently large range of loads. The Young’s modulus of this material is about 2.1 GPa, so its value is of the same order as the constants determined for printed structures. This enables the correct cooperation of model components made of both types of materials.

**Figure 11:**
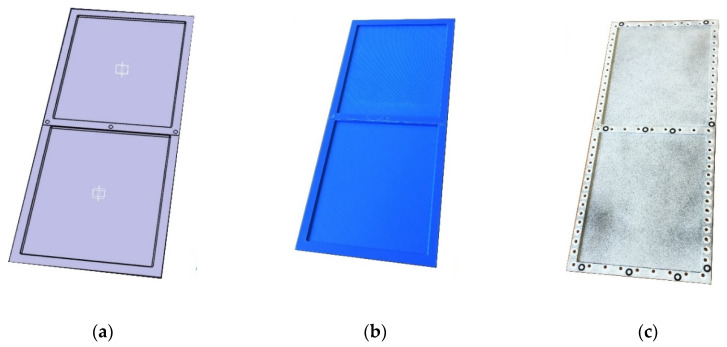
View of the torsion box wall: (**a**) CAD virtual model; (**b**) printed model before processing; (**c**) printed model prepared for assembly and optical scanning.

**Figure 12:**
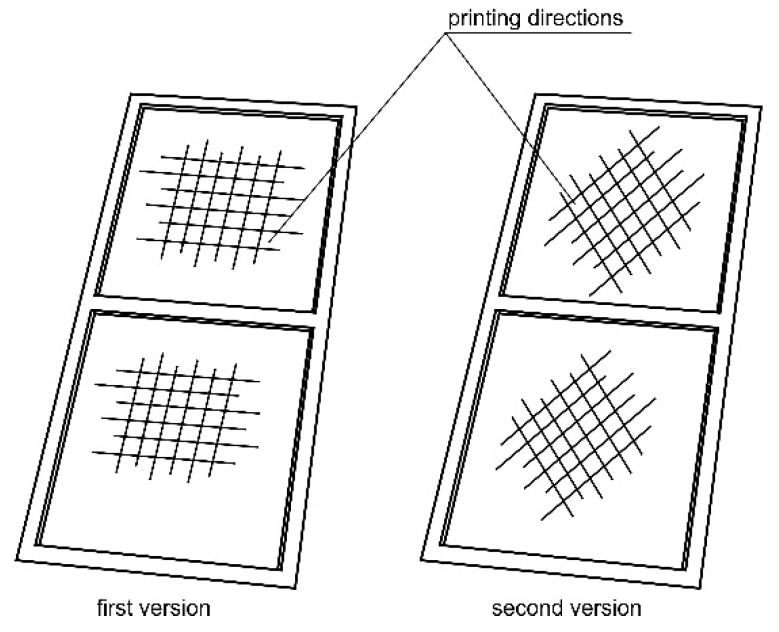
Model printout structure diagram.

The wall of the torsion box was modeled in the 3D CAD software CATIA v5 (Dassault Systèmes, Vélizy-Villacoublay, France) and was made using the 3D printing technique, in the form of a thin plate with integral reinforcements in the areas of the intended bolted connections (Figure 11). Two versions of the wall model were made with different printing directions. The areas of smaller thickness in both cases were made of two layers with mutually perpendicular directions for the material application. In the first model, these directions corresponded to the directions of the wall edges, while in the second model they were oriented in relation to them at an angle of 45 degrees (Figure 12).

**Figure 13:**
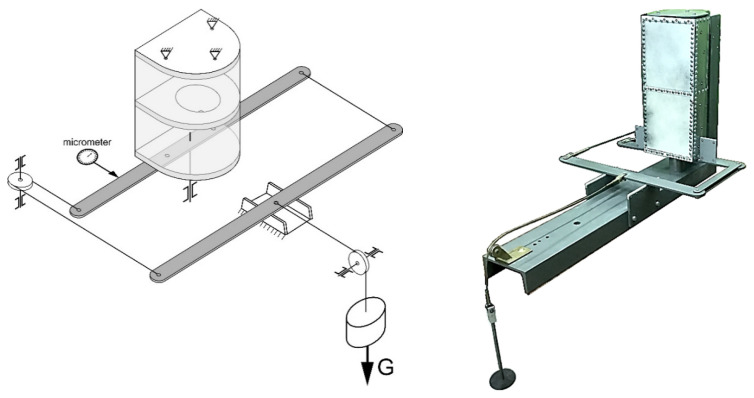
Scheme and general view of the test stand with the mounted model.

The complete models were fixed on a test stand and subjected to twisting. The upper frame of the model was attached to the stand frame with three bolts. A single bolt in the axis of rotation was used in the lower frame as a bearing. Displacement measurements were conducted using the Aramis optical scanner. Moreover, the construction of the stand made it possible to measure the angle of torsion of the tested system using a micrometer (Figure 13). The load was conducted using the gravity method and displacement values were recorded for the established equilibrium states of the system.

In the next stage of research, numerical calculations were conducted with the use of the MSC PATRAN/MSC MARC commercial software (MSC Software, Newport Beach, CA, USA), based on the finite element method.

For the spar wall, a material model representing a layered laminate was adopted and the mechanical properties were defined for each of the layers, assuming the previously marked physical constants and determining the layer thickness and orientation of the printed fibers. Therefore, the material model for the system made with 3D printing technology was analogous to the models used for the layered composites.

Due to the nature of the experiment, during which post-critical deformations of the torsion box wall occurred, it was necessary to use nonlinear analyses [34,35].

In its assumptions, the nonlinear numerical analysis involving the finite element method is based on the gradual nature of the solution and the determination of the static equilibrium state in each subsequent step [36], which necessitates solving the matrical equation of residual forces according to Equation (2):

(2) r(u,Λ)=0

where u is a state vector containing displacement components defining the current state of the system, Λ is a so-called control matrix containing parameters related to the current load, while r. is a residual vector containing unbalanced components of internal forces.

For Clapeyron systems with fixed load parameters, the residual vector is defined as the total potential energy gradient of the system Π according to Equation (3):

(3) $$r=\frac{\partial \Pi }{\partial u}.\;$$

Equation (2) can also be presented in the following form of Equation (4):

(4) p(u)=f(u,Λ)

where **p** is a matrix containing internal forces corresponding to the current deformation state and **f** is an external forces vector, which can be presented in the following form:

(5) $$p=\frac{\partial U}{\partial u},\;\;\;\;f=\frac{\partial P}{\partial u}$$

where **p** is the elastic strain energy and **u** is the external load work. The system total potential energy can be expressed:

(6) Π=U−P

The system stiffness matrix **K**, also called the tangent matrix, corresponding to its current temporary state is defined as the derivative of the residual vector towards the state vector according to Equation (7):

(7) $$K=\frac{\partial r}{\partial u}$$

By obtaining the derivative of the vector **r** relative to the control parameters, a control matrix, also called a load matrix, can be determined:

(8) $$Q=-\frac{\partial r}{\partial \Lambda }$$

A quantity known as the pseudo-time is used to describe the progress of a nonlinear numerical task t, i.e., a dimensionless parameter linking the current load with the current state of the structure according to Equation (9):

(9) u=u(t),Λ=Λ(t)

The derivative of the component r of the residual vector in relation to **t** has the form:

(10) $${\dot{r}}_{i}=\frac{\partial r_{i}}{\partial u_{j}}{\dot{u}}_{j}+\frac{\partial r_{i}}{\partial \Lambda_{j}}{\dot{\Lambda }}_{j}$$

where:

(11) $$\dot{r}=\frac{\partial r}{\partial t}$$

From dependences (7) and (8):

(12) $$\dot{r}=K\dot{u}-Q\dot{\Lambda }$$

The second derivative of the residual vector **r** relative to the parameter t has the form:

(13) $$\dot{r}=K\ddot{u}+\dot{K}\dot{u}-Q\ddot{\Lambda }-\dot{Q}\dot{\Lambda }$$

where matrices $\dot{K}$ and $\dot{Q}$ are:

(14) $$\dot{K}=\frac{\partial K}{\partial t},\dot{Q}=\frac{\partial Q}{\partial t}$$

For most numerical nonlinear problems, the control matrix components can be expressed as functions of a single parameter λ, called the control parameter. Then, Equation (2) takes its form according to Equation (15):

(15) r(u,λ)=0

In modern software, in addition to the prognostic phase, which determines the change in the state of the structure in the next step of the solution, the correction phase is also used, which consists of filling an additional matrix equation, called the growth control equation or the constraints equation, according to Equation (7):

(16) $$c\left(\Delta u_{n},{\Delta \lambda }_{n}\right)=0$$

where the size $\Delta u_{n}$ and ${\Delta \lambda }_{n}$ are defined with Equation (8):

(17) $$\Delta u_{n}=u_{n+1}-u_{n},{\;\Delta \lambda }_{n}=\lambda_{n+1}-\lambda_{n}$$

and are the increments corresponding to the transition of the structure from state n to state n + 1.

In the case of the nonlinear numerical analyses presented in this study, the Newton–Raphson prognostic method was used with a correction strategy based on state control [37].

**Figure 14:**
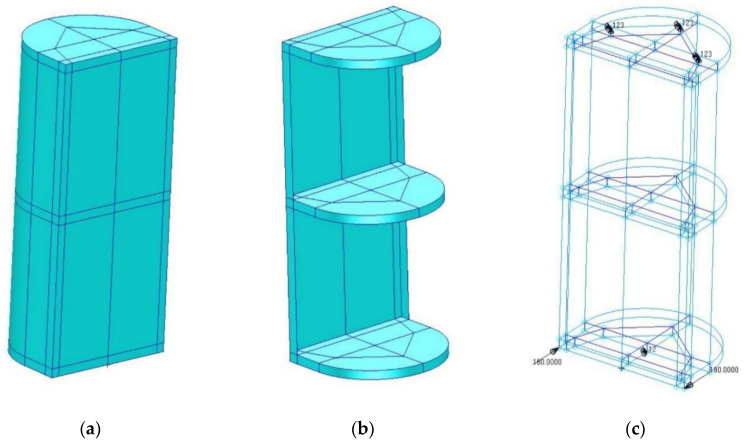
FEM geometric model: (**a**) general view; (**b**) model with a partially hidden shell; (**c**) model with visible edge conditions and loading.

In order to numerically represent the conditions of the experiment, a surface–solid model was used (Figure 14).

**Figure 15:**
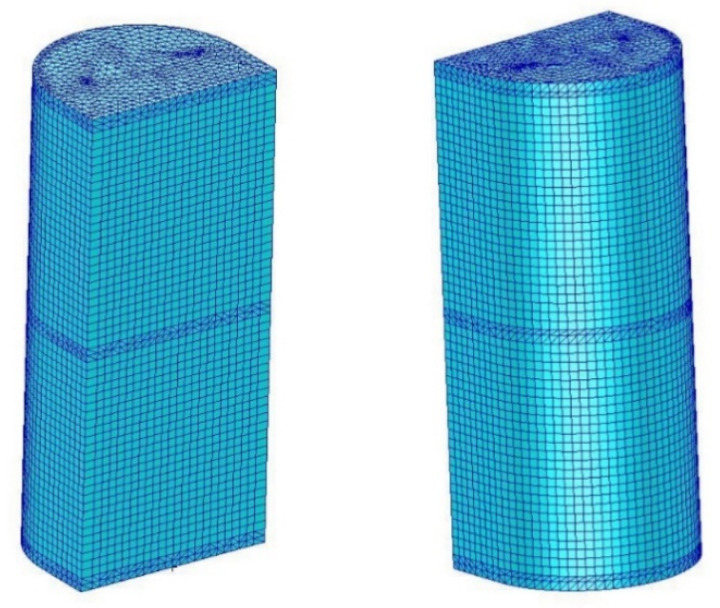
Finite elements mesh view.

The finite element mesh has a hybrid nature (Figure 15). In the case of surface objects, four-node, thin-walled shell elements with a linear shape function were used. In the case of frames that were not the subject of analyses, a mesh consisting of four-node tetrahedral elements was used.

The mesh density was determined through a series of trial analyses. The final form of the mesh of the skin included 4641 shell elements and 4721 corresponding nodes. The mesh of the frames included 24,260 solid elements and 9187 nodes.

## 3. Results

### 3.1. Experimental Research

In the first phase of experimental research, the physical constants for the printed structure were determined. The obtained values of the constants are presented in Table 1.

The obtained results from the mechanical data for PLA (Verbatim Ltd, Egham, UK) correspond with the good agreement with the recently published papers. As an example, it can be noted that Brischetto and Torre [38], from an extensive experimental set of analyses, obtained Young’s modulus values in the range of 2.4 GPa to 2.7 GPa and tensile stress values ranging from 59 MPa to 64 MPa for specimens printed in the direction of stretching. Raj et al. [39], based on tension tests, obtained maximum stress values for a PLA specimen in the range of 47–50 MPa and Young’s modulus values ranging from 2.8–3.4 MPa. The results published by Yao et al. [40] showed that regarding 3D printing in vertical directions, the values for the modulus of elasticity can vary from 1.5 GPa to 2.2 GPa and maximal stress changes can vary from 23 MPa to 54 MPa.

**Figure 16:**
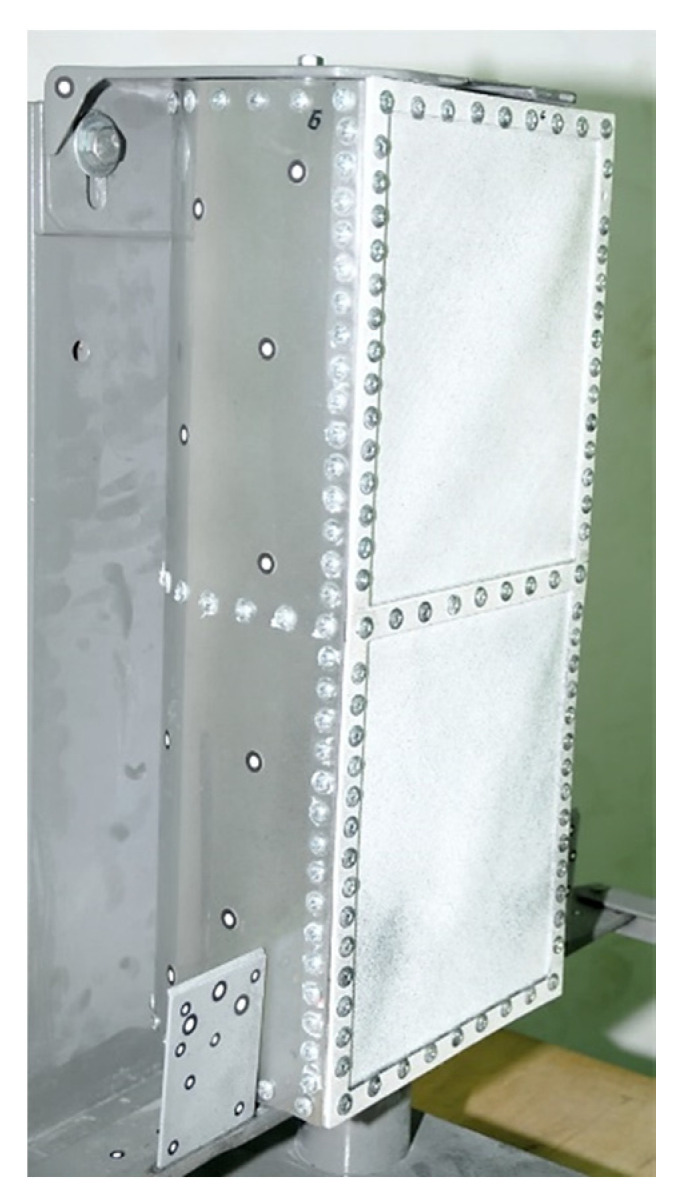
View of the wall deformation at the maximum load value (156 N).

Next, both versions of the torsion box model were experimented with. As a result of twisting, in both cases, diagonal folds were formed on the spar walls (Figure 16). This form of post-critical deformation results from the formation of tension fields within the wall segments. Unfortunately, the conditions of the experiment did not enable the same deformation distributions to be obtained, which could be attributed to imperfections of the model structure. As a result of resetting the backlash in the bolted connections during the trial loading of the models, initial stresses appeared, as a result of which the tangential stress distributions deviated from the ideal distribution resulting from the static in the system.

**Figure 17:**
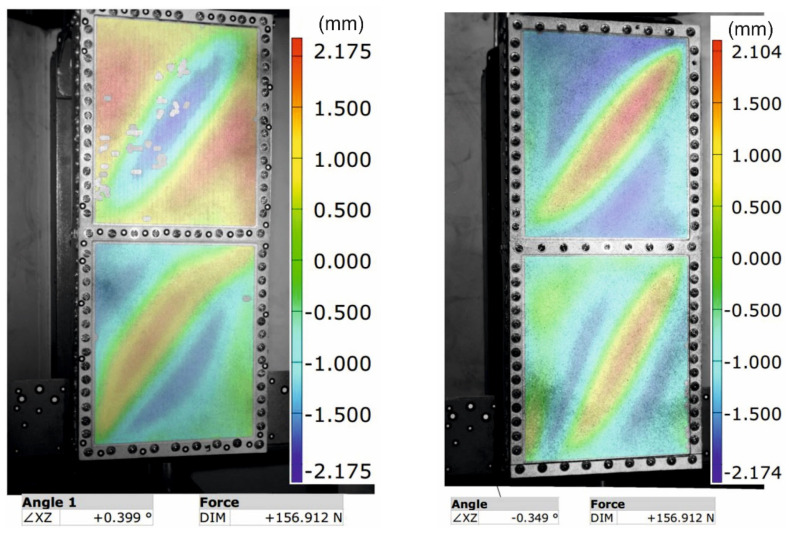
Results of measurements of the displacement component in the normal direction to the wall plane, determined with an optical scanner.

**Figure 18:**
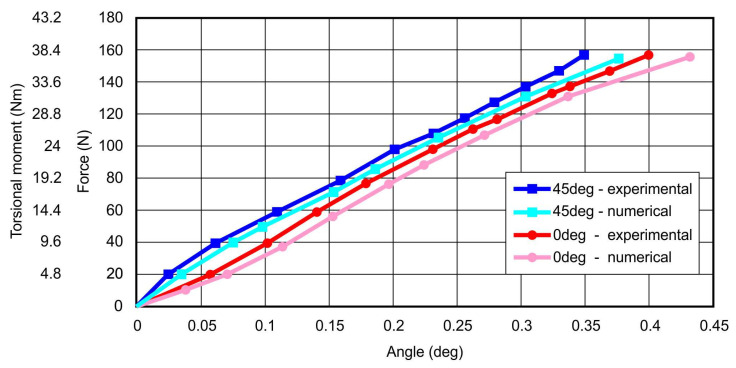
Set of representative equilibrium paths.

As a result of experimental studies on the torsion box models, the displacement distributions of the spar wall in the perpendicular direction to its plane were obtained (Figure 17), as well as the relationship between the total torsion angle and the value of the applied load, on the basis of which representative equilibrium paths were crossed, representing the relationship between the structure torsion angle and the torsional moment (Figure 18).

### 3.2. Nonlinear Numerical Analyses

On the basis of the results of nonlinear numerical analyses, representative equilibrium paths of both analyzed structures were determined, which were compared with the corresponding relationships obtained during the experiment (Figure 18).

The presented representative equilibrium paths are characterized by satisfactorily high convergence. The differences between angles values obtained for the highest load levels do not exceed 7.5%, and for the area of loads below 80% of the maximum loads do not exceed 5%.

**Figure 19:**
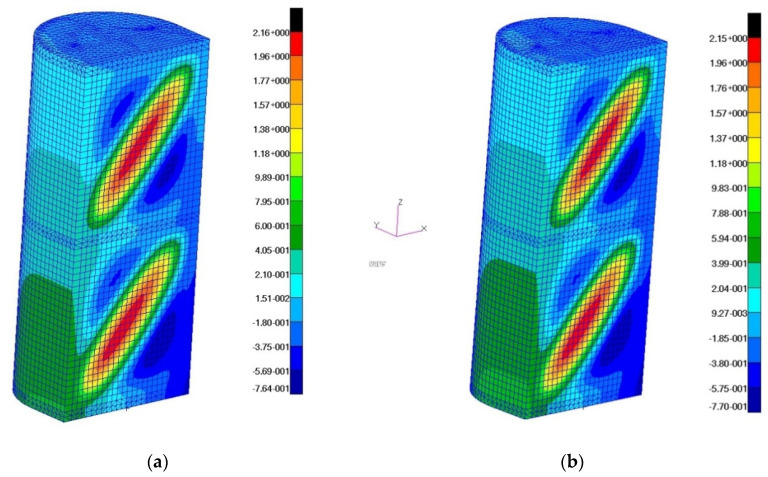
Distributions of displacements in the direction of the *Y*-axis of the reference system: (**a**) a wall with a fiber orientation corresponding to the X, Z directions; (**b**) a wall with fiber orientation at an angle of 45 degrees in relation to the X, Z directions.

The model deformation distributions for the maximum value of the applied load were also obtained (Figure 19).

**Figure 20:**
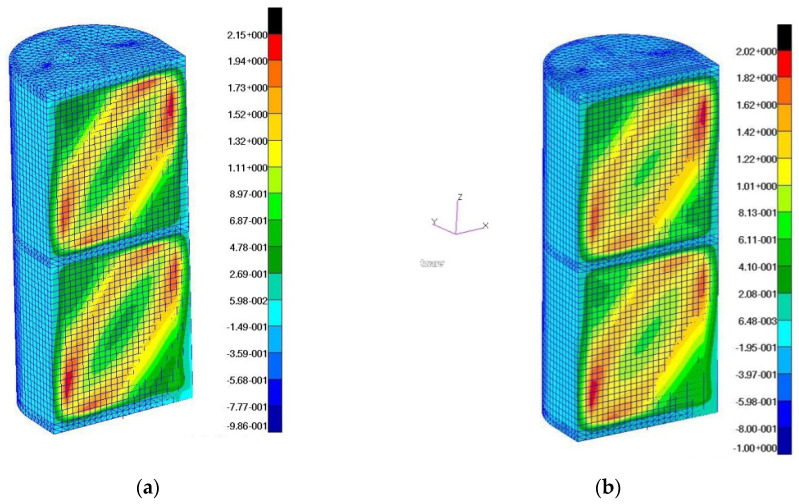
Distributions of tangential stresses: (**a**) a wall with fiber orientation corresponding to the X, Z directions; (**b**) a wall with fiber orientation at an angle of 45 degrees to the X, Z directions.

**Figure 21:**
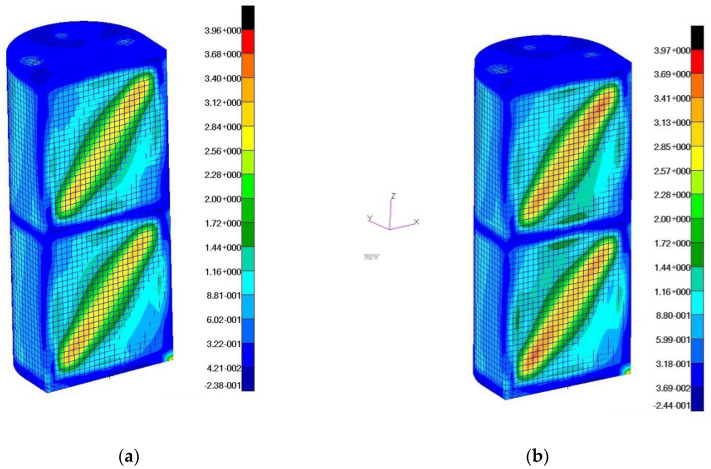
Distributions of reduced stress according to the σmax hypothesis: (**a**) a wall with the fiber orientation corresponding to the X, Z directions; (**b**) a wall with the fiber orientation at an angle of 45 degrees to the X, Z directions.

The tangential stress distributions corresponding to the above deformation states (Figure 20) and the reduced stress distributions according to the σ_max_ hypothesis (Figure 21) were also determined.

## 4. Discussion

The overriding idea behind the presented considerations and the conducted research was to determine the purpose of using an analogous model, as in the case of layered composites, in reference to the thin-walled structure created with 3D printing technology from a biodegradable polymer, which was polylactic acid. The very nature of 3D printing poses limitations in terms of obtaining material homogeneity (Figure 1). While observing the cross-section of a single printed layer, clear differences were observed between the cross-sectional surfaces of the fibers and the joints connecting them. Hence, there is an orthotropy in terms of mechanical properties. This was confirmed by experiments aimed at determining the appropriate physical constants. Naturally, the exemplary difference between Young’s modulus sizes along and across the direction of the fibers is not as significant as for some composites; however, multiple experiments using a series of samples confirmed this difference to be about 15%, which may affect the behavior of the complete structure under various load conditions. Therefore, the concept of considering the directional properties of individual print layers in detail when creating calculation models seems legitimate.

On the other hand, the validity of the experimental determination of the physical constants for the examined structure seems to be advisable, because as found herein, the values determined during the described experiments significantly differ from the values given by the published sources. In reference to the structure made of a polymer using the hot formatting technique, it is difficult to make assumptions about the universal nature of the results obtained for a specific device with the use of specific printing parameters (e.g., diameter of a material dispensing nozzle). Additionally, the discrepancy in mechanical properties between objects made of different batches of raw material from different manufacturers cannot be excluded. The presented methodology for determining physical constants should, therefore, be treated as a proposal for a certain type of procedure, with the recommendation of using it each time when testing polymer printed structures.

The validity of the thesis regarding the necessity to use the above-described method of numerical modeling for printed structures was confirmed by the results of experiments using the torsion box model with a wall made of polylactide. It should be emphasized that the nature of the work for both examined structures under the conditions of post-critical loads was very similar, as a result of the appearance of a physical imperfection in the form of a tension field in both wall segments. Therefore, no noticeable differences in the nature of deformation were expected; however, a detailed comparative analysis of the displacement distribution (Figure 17) enabled clear differences to be observed, both in terms of quality and quantity. Nonlinear numerical analyses confirmed this difference, although the values of the calculated displacements were not fully consistent with those determined experimentally. It should be emphasized once again that the conditions of the experiment introduced certain disturbances in the form of initial geometric imperfections, while the numerical model was based on an idealized geometry. Therefore, it was concluded that the determinant of the satisfactory compliance of the numerical calculations with the experiment is the similarity of the representative equilibrium paths, representing the state of the structure as determined by the total angle of its twist and load. As shown in the graph (Figure 18), the discrepancies between the specific characteristics obtained experimentally and numerically were very small. Numerical models showed a slightly lower stiffness than their real equivalents, which may be due to some inaccuracy in the range of determining the physical constants of the material. Some errors could also be introduced with simplified numerical mapping of the edge conditions. Ultimately, however, considering the very slight differences in the course of the above characteristics, the convergence of the results can be regarded as satisfactory.

Both experimental research and numerical analyses have also confirmed the clear influence of the orientation of the fibers of a printed structure on the nature of its deformation. Taking into account the above, although relatively small differences between the physical constants were observed along and across the fibers, and while the final discrepancies in terms of the nature and size of the deformation were also small, these factors cannot be considered negligible, especially in the case of objects as sensitive as thin-walled aircraft structures, which must meet restrictive standards.

## 5. Conclusions

The presented research has shown that the elements of load-bearing structures made using 3D printing techniques and from a biodegradable material, namely polylactic acid, can be used for ultra-light unmanned aerial vehicles due to the materials relatively favorable mechanical properties. However, it should be emphasized that they show a number of characteristic features influencing the stiffness and strength of the system. These features depend on the print parameters and individual properties of the printing device, as well as the batch and origin of the used raw material.

On the other hand, certified aircraft structures are particularly demanding in terms of the need to meet strict standards, and are subject to detailed control during operation. At the design stage, it is, therefore, necessary to conduct detailed calculations of these types of structures based on correct structural models and taking into account the actual mechanical properties of the printed components.

In light of the above observations, one can, therefore, formulate a thesis that in relation to the above-mentioned structures, it is recommended to experimentally determine the physical constants of the printed elements of the devices and raw materials used for the project. It also seems justified to create a computational model for laminates whose individual layers are treated in an analogous manner to fibrous composites.

The presented considerations allow the proposal of the implementation of the above-mentioned stages of the process, which were based on a relatively cheap experiment. The described imperfections in terms of the results of the experiment conducted on the torsion box model lead to the conclusion that in further research it is necessary to improve the model, for example by using alternative connection solutions between the components.

## Figures and Tables

**Table 1:** Values of physical constants for the printed structure.

	Young’s Modulus, GPa	Tensile Stress, MPa	Poisson’s Ratio ν12	Shear Modulus, MPa
Direction 1 ^1^	2.71 ± 3%	50 ± 8%	0.328 ± 5%	1.24 ± 7%
Direction 2	2.31 ± 4%	30 ± 10%		

^1^ Accordant with the direction of stretching.
